# Promoters Are Differentially Sensitive to N-Terminal Mutant Huntingtin-Mediated Transcriptional Repression

**DOI:** 10.1371/journal.pone.0041152

**Published:** 2012-07-18

**Authors:** Matthew Hogel, Robert B. Laprairie, Eileen M. Denovan-Wright

**Affiliations:** Laboratory of Molecular Neurobiology, Department of Pharmacology, Dalhousie University, Halifax, Nova Scotia, Canada; Institut de Génomique Fonctionnelle, France

## Abstract

Huntington’s disease (HD) is a neurodegenerative disorder caused by the inheritance of one mutant copy of the *huntingtin* gene. Mutant huntingtin protein (mHtt) contains an expanded polyglutamine repeat region near the N-terminus. Cleavage of mHtt releases an N-terminal fragment (N-mHtt) which accumulates in the nucleus. Nuclear accumulation of N-mHtt has been directly associated with cellular toxicity. Decreased transcription is among the earliest detected changes that occur in the brains of HD patients, animal and cellular models of HD. Transcriptional dysregulation may trigger many of the perturbations that occur later in disease progression. An understanding of the effects of mHtt may lead to strategies to slow the progression of HD. Current models of N-mHtt-mediated transcriptional dysregulation suggest that abnormal interactions between N-mHtt and transcription factors impair the ability of these transcription factors to associate at N-mHtt-affected promoters and properly regulate gene expression. We tested various aspects of the current models using two N-mHtt-affected promoters in two cell models of HD using overexpression of known N-mHtt-interacting transcription factors, promoter deletion and mutation analyses and *in vitro* promoter binding assays. Consequently, we proposed a new model of N-mHtt-mediated transcriptional dysregulation centered on the presence of N-mHtt at promoters. In this model, N-mHtt interacts with multiple partners whose presence and affinity for N-mHtt influence the severity of gene dysregulation. We concluded that simultaneous interaction of N-mHtt with multiple binding partners within the transcriptional machinery would explain the gene-specificity of N-mHtt-mediated transcriptional dysregulation, as well as why some genes are affected early in disease progression while others are affected later. Our model explains why alleviating N-mHtt-mediated transcriptional dysregulation through overexpression of N-mHtt-interacting proteins has proven to be difficult and suggests that the most realistic strategy for restoring gene expression across the spectrum of N-mHtt affected genes is by reducing the amount of soluble nuclear N-mHtt.

## Introduction

Huntington’s disease (HD) is an autosomal dominant neurodegenerative disease [1]. HD is recognized primarily as a movement disorder, however patients suffering from HD will also experience cognitive impairments and psychiatric disturbances [2]. Symptoms of HD normally do not present until the fourth or fifth decade of life, and following onset the symptoms will increase in severity over the next fifteen to twenty years until the patient succumbs to the illness [3]–[6]. The mutant allele of the *huntingtin* gene is defined as having greater than 36 repeats in a polymorphic CAG repeat region in exon 1 [7]. Translation of the mutant allele produces a protein containing an abnormally long polyglutamine (polyQ) repeat region located near the N-terminus. Cleavage of the huntingtin protein (Htt) resulting in the release of a smaller N-terminal fragment has been shown to occur in unaffected individuals [8], however, the presence of an expanded polyQ region in the mutant huntingtin protein (mHtt) results in increased cleavage [9]. The cleaved N-terminal fragment of the mutant huntingtin protein (N-mHtt) translocates to, and accumulates in, the nucleus [10]. Both the formation of N-mHtt and its accumulation in the nucleus have been associated with cellular pathology and with the progression of the disease [11], suggesting that it is the nuclear form of N-mHtt that is most toxic to the cell.

Expression of mHtt leads to a variety of changes in cellular function, including changes in the proteasomal degradation pathway [12], autophagy [13], apoptosis [14], mitochondrial function [15], neurotrophic support [16], cholesterol biosynthesis [17], intercellular signalling [18], and transcriptional regulation [19]. Some of these changes may result directly from the expression of mHtt, while others may be compensatory changes. The earliest detectable changes are most likely to result directly from the presence of mHtt. One change that is consistently seen in human patients, as well as in all animal models of HD, and that is among the earliest detectable changes is the altered expression of a subset of genes in specific cells in the body [20], [21]. Given the correlation between symptoms of HD and the presence of N-mHtt in the nucleus [10], [11], and given the connection between transcription and the downstream changes that occur in the cell during the progression of HD, transcriptional dysregulation may represent an initiation point in the cascade of changes that occurs following expression of mHtt [21]. If these assumptions are correct, alleviating N-mHtt-mediated transcriptional dysregulation may provide a mechanism to slow the progression of HD.

The current theories regarding how N-mHtt inhibits transcription are predicated on known interactions between N-mHtt and proteins involved in transcriptional regulation. N-mHtt interacts with proteins involved in the regulation of chromatin structure [22]–[24], with gene-specific transcription factors [25]–[27], co-activators [24], [28], and with members of the general transcription machinery [29], [30]. *In vitro* transcription assays, using purified transcription factors and recombinant N-mHtt, have demonstrated that N-mHtt is able to inhibit transcription in the absence of chromatin [30]. Thus, although impaired chromatin folding may contribute to aspects of transcriptional dysregulation, N-mHtt is able to inhibit transcription through direct actions with transcription factors or the general transcription machinery. The goal of this study was to build on the understanding of how N-mHtt impairs transcription through actions with gene-specific transcription factors and the general transcriptional machinery. The current theories of N-mHtt-mediated transcriptional dysregulation were tested using the human cytomegalovirus immediate early gene (CMV) promoter and the herpes simian virus thymidine kinase (TK) promoter as model promoters. Specifically, we attempted to determine whether a specific portion of a N-mHtt-affected promoter was required by N-mHtt to inhibit transcription. We attempted to determine whether known N-mHtt-interacting transcription factors were involved in the inhibition of CMV or TK activity, and whether transcriptional dysregulation could be alleviated through overexpression of specific interacting proteins. We also attempted to determine if N-mHtt impaired transcription by sequestering proteins away from the DNA, or whether N-mHtt was directly associated with promoter-bound complexes. Lastly, we sought to investigate the relationship between the amount of N-mHtt expressed in a cell and the susceptibility of the CMV and TK promoters to transcriptional dysregulation.

## Results

**Figure 1:**
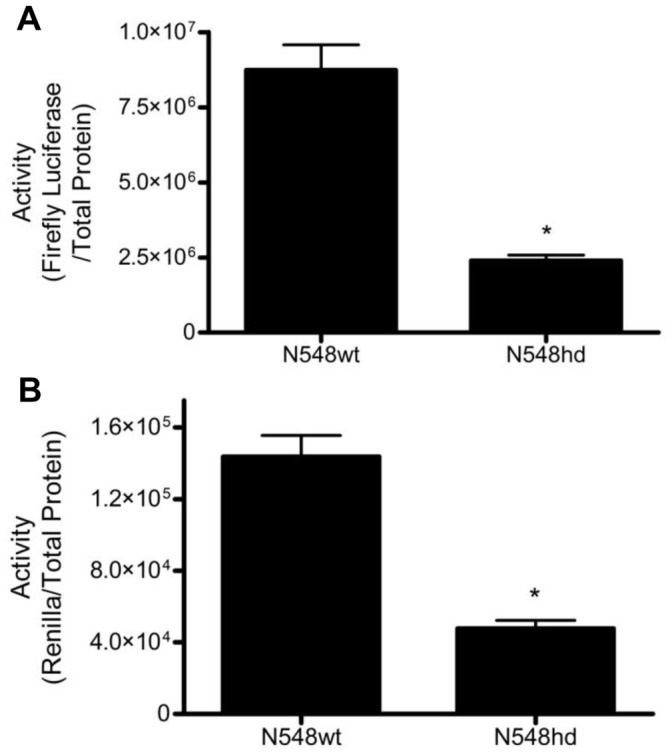
CMV and TK activity were decreased in N548hd cells. CMV-driven firefly luciferase activity (A) and TK-driven *Renilla* luciferase activity (B) were normalized to total protein in cell lysates. * *P<*0.05 relative to N548wt as determine by two-tailed *t*-test. Data are shown as mean ± S.E.M. n = 8 per cell type.

Reporter plasmids under the control of the CMV and TK promoters were co-transfected into both N548wt and N548hd cells. The luciferase activity driven by the two reporter plasmids was quantified 24 h following transfection using the Dual-Luciferase Reporter (DLR) Assay. Transcription driven by both the CMV and the TK promoters was significantly lower in N548hd cells relative to activity in N548wt cells (Fig. 1). This result suggested that the CMV and TK promoters, when expressed in the N548 cell lines, provide a model for studying the inhibitory effects of N-mHtt on transcription, as well as for comparing and contrasting the effect of N-mHtt on different promoters.

**Figure 2:**
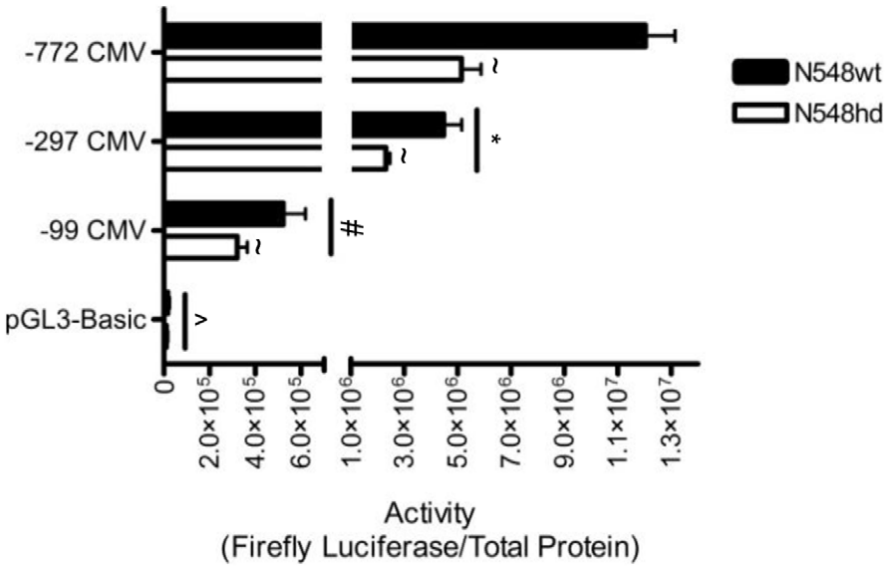
Transcription driven by the−99 CMV promoter was inhibited by N-mHtt. The −772 CMV promoter was sequentially deleted to 297 (−297 CMV) and 99 (−99 CMV) bp upstream of the transcription start site and promoters were inserted into the pGL3-Basic reporter plasmid. Activity of these plasmids in N548wt and N548hd cells is shown normalized to total protein. * *P<*0.05 relative to −772 CMV. # *P<*0.05 relative to −772 CMV and −297 CMV. ∧ *P<*0.05 relative to −772 CMV, −297 CMV and −99 CMV ∼ *P<*0.05 relative to N548wt cells as determined by two-way ANOVA followed by a Tamhane’s T2 post-hoc test for unequal variance to analyze effect of promoter deletion, and one-tailed *t*-test to analyze cell-type effect. Data are shown as mean ± S.E.M. n = 8 per data set.

To determine if the region of the CMV promoter required for transcriptional repression in the presence of N-mHtt could be identified, fragments of the CMV promoter spanning 772, 297 and 99 bp 5′ to the transcription start site (−772 CMV, −297 CMV, and −99 CMV) were transfected into N548wt and N548hd cells. The sequential deletion of the CMV promoter resulted in progressively lower luciferase activity (Fig. 2), indicating that activator elements reside in the region between 100 and 772 bp on the CMV promoter. Activity of the −99 CMV promoter was significantly higher than the background activity produced by empty pGL3-Basic plasmid, which indicated that deletion of the CMV promoter to the 99 bp proximal to the transcription start site did not completely eliminate transcriptional activity. With regards to the inhibitory effects of N-mHtt on transcription driven by the CMV promoter, luciferase activity was significantly lower in N548hd cells than in N548wt cells for all of the CMV promoter deletion fragments tested. There was no difference in the activity of the pGL3-Basic control plasmid in N548hd cells compared to N548wt cells, suggesting that N-mHtt-mediated repression of luciferase activity was specific to the CMV promoter. As the sequential reduction of the CMV promoter did not alleviate N-mHtt-mediated transcriptional dysregulation, it appeared that N-mHtt-mediated repression of the CMV promoter was localized to the region within 99 bp of the transcription start site.

**Figure 3:**
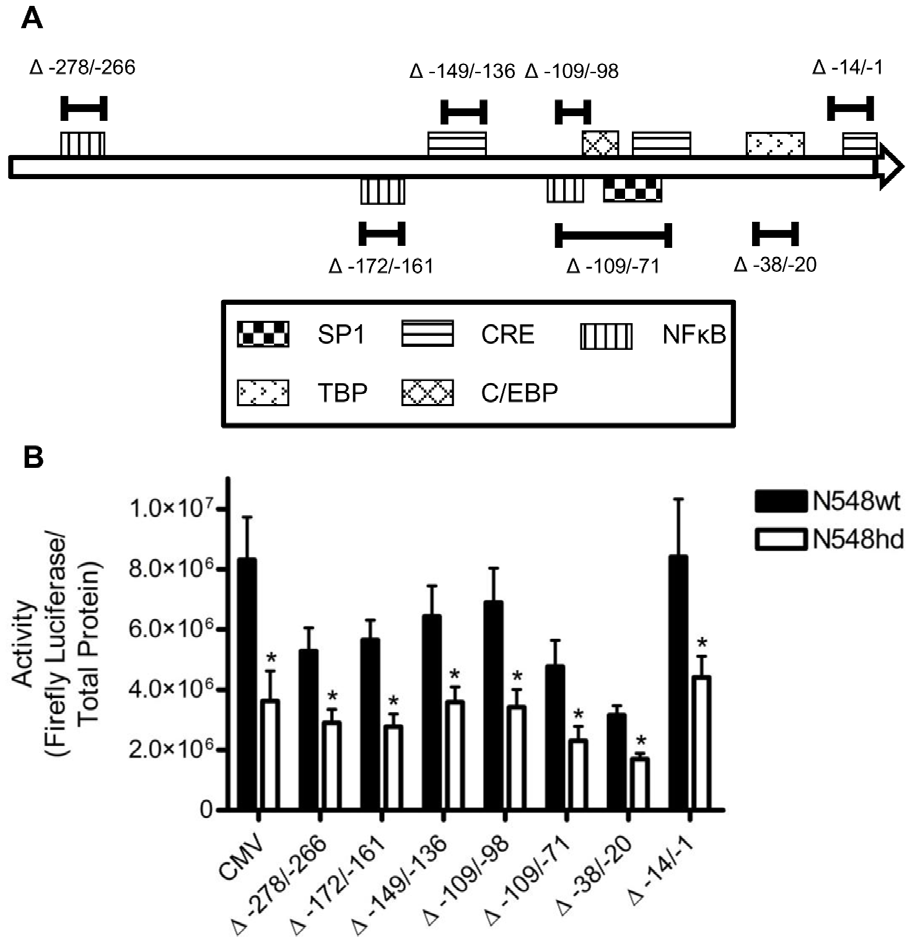
Mutation of specific DNA sequences did not relieve N-mHtt-mediated transcriptional repression of the CMV promoter. (A) Putative binding sites for transcription factors of interest on the −297 CMV promoter. Binding sites for Sp1, TBP, CREB, C/EBP, and NFκB present on the CMV promoter within the 297 bp upstream of the transcription start site are shown. Bars indicate the region of the promoter mutated using linker scanning mutagenesis, while the numbers indicate the location of these mutations, relative to the transcription start site. (B) The DNA sequences on the CMV promoter indicated (relative to the transcription start site) were mutated using linker scanning mutagenesis and inserted into pGL3-basic reporter plasmid. Activity of these plasmids in N548wt and N548hd cells is shown normalized to total protein. * *P<*0.05 relative to activity of the same plasmid in N548wt cells, as determined by one-tailed *t-*test. Data are shown as mean ± S.E.M. n = 8 per data set.

One of the hypothesized mechanisms regarding how N-mHtt inhibits transcription suggests that N-mHtt interacts with and sequesters gene-specific transcription factors. If transcription driven by the CMV promoter relied on the function of N-mHtt interacting transcription factors, functional impairment of that factor resulting from its interaction with N-mHtt could explain the decreased CMV activity detected in N548hd cells. The 227 bp of CMV promoter upstream of the transcription start site were analyzed using MatInspector software to identify putative transcription factor binding sites of proteins known to interact with N-mHtt. This CMV promoter fragment contained three putative NF-κB binding sites, three putative CREB response element (CRE) binding sites, one putative Sp1 binding site, one putative binding site for CCAAT-enhancer binding protein (C/EBP) and one putative TATA binding protein (TBP) binding site (Fig. 3A). Each site was eliminated using linker-scanning mutagenesis to determine whether they were involved in N-mHtt-mediated transcriptional repression of the CMV promoter. It was hypothesized that if a specific transcription factor binding site was required by N-mHtt to inhibit CMV activity, disruption of that binding site would eliminate the difference in CMV activity in N548hd cells and N548wt cells. Each promoter mutant had significantly lower transcriptional activity in N548hd cells compared to N548wt cells (Fig. 3B). Therefore, none of the tested transcription factors that bound to their respective binding sites was solely responsible for transcriptional repression of the CMV promoter in the presence of N-mHtt. Since sequential deletion of the CMV promoter demonstrated that N-mHtt required the minimally active region of the promoter to inhibit transcription, the inability to alleviate transcriptional inhibition of the CMV promoter by mutating the binding sites of candidate N-mHtt-interacting transcription factors suggested that N-mHtt might inhibit transcription by interfering with the general transcription machinery.

**Figure 4:**
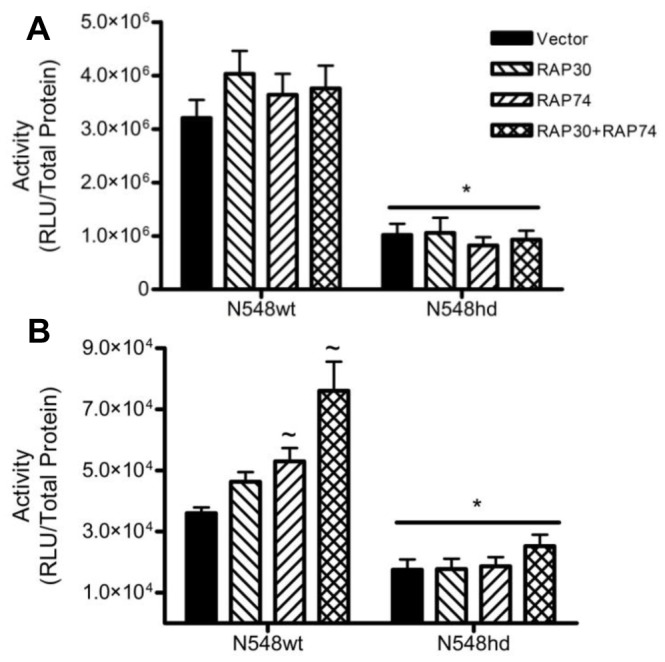
Overexpression of the components of TFIIF did not recover N-mHtt-mediated transcriptional repression. N548wt and N548hd cells were transfected with reporter plasmids driven by either the CMV (A) or TK (B) promoters and either an empty expression plasmid or ones driving production of human RAP30 or human RAP74 protein. Luciferase activity was normalized to total protein. * P<0.05 relative to N548wt as determined by a two-way ANOVA. ∼ *P<*0.05 relative to vector control within the same cell type as determined by two-tailed *t-*tests. Data are shown as mean ± S.E.M. n = 8 per data set.

A study by Zhai *et al.* (2005) demonstrated that N-mHtt interacts with the RAP30 subunit of the general transcription factor IIF (TFIIF), and that the N-mHtt/RAP30 interaction was involved in transcriptional repression of a 75 bp fragment of the D_2_ dopamine receptor promoter [30]. We hypothesized that if an interaction between N-mHtt and RAP30 led to inhibition of CMV or TK activity in N548hd cells, overexpression of RAP30 would alleviate this transcriptional repression. Plasmids driving the transcription of human RAP30 cDNA, or its empty vector control, were transfected into N548wt and N548hd cells with reporter plasmids driven by the CMV and TK promoters. CMV and TK activity were assayed 24 h following transfection. Overexpression of RAP30 failed to increase CMV or TK activity in N548hd cells (Fig. 4). The study by Zhai *et al.* (2005) showed that the N-mHtt interacted at the region of RAP30 required for its association with its TFIIF binding partner RAP74 [30]. If N-mHtt was competing with RAP74 for association with RAP30 in N548hd cells, we hypothesized that overexpression of RAP74 would shift this competition to favour formation of TFIIF. Plasmids driving the expression of RAP74 cDNA, or its empty vector control, were transfected into N548wt and N548hd cells along with CMV and TK reporter plasmids. Overexpression of RAP74 led to a significant increase in TK activity in N548wt cells, suggesting either that RAP74 levels were limiting in N548wt cells, or that the N-terminal fragment of Htt expressed in N548wt cells was inhibiting TK activity using a mechanism that involved TFIIF. Overexpression of RAP74 failed to increase either CMV or TK activity in N548hd cells (Fig. 4). Two hypotheses may explain the inability of RAP30 or RAP74 overexpression to alleviate N-mHtt-mediated transcriptional repression. First, because N-terminal Htt is expressed under the control of a viral promoter in the N548wt and N548hd cells, the levels of N-mHtt in N548hd cells may have been in excess of RAP30 or RAP74. Second, N-mHtt may inhibit the function, and not the formation, of TFIIF.

If levels of RAP74 were limiting, and N-mHtt interacted with RAP30 in a manner that did not inhibit the formation of TFIIF, overexpression of RAP30 or RAP74 on their own would not restore transcription in the presence of N-mHtt. In an attempt to increase the amount of functional TFIIF in N548hd cells, plasmids driving the expression of RAP30 and RAP74 cDNA were co-transfected into N548wt and N548hd cells along with reporter plasmids driven by the CMV and TK promoters. TK activity was significantly increased in N548wt cells following overexpression of both RAP30 and RAP74 (Fig. 4). This result was not seen in the presence of N-mHtt. Simultaneous overexpression of RAP30 and RAP74 failed to increase transcription driven by the CMV promoter in both N548wt and N548hd cells (Fig. 4).

**Figure 5:**
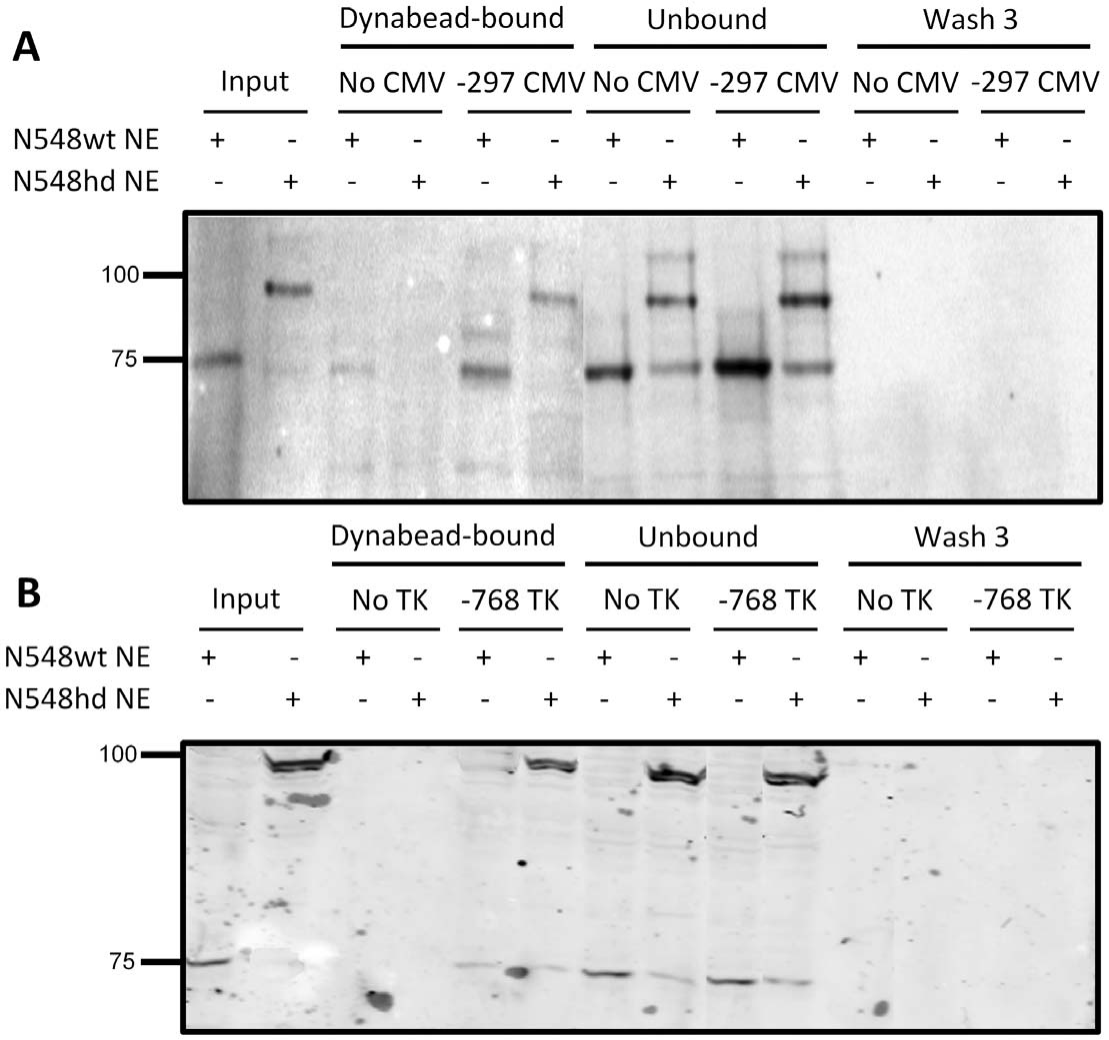
N-terminal Htt was present in the protein complement that bound to the CMV and TK promoters. Nuclear extract isolated from N548wt and N548hd cells was incubated with −297 CMV promoter **(A)** or −768 TK promoter **(B)** attached to magnetic beads (−297 CMV/−768 TK) or to magnetic beads with no attached DNA (No CMV/No TK). After a 30 m incubation the reactions were washed three times and the protein complexes that remained bound were collected. The protein from the bound and unbound fractions, as well as the protein in the third wash were fractionated on a SDS-PAGE gel. MAB2166 antibody, which is specific to N-mHtt, was used for western blotting. Numbers on the left of the figure represent relative mobility of molecular weight size markers in kDa.

The previous result suggested either that the amount of N-mHtt generated in the N548 cell lines could not be overcome through overexpression of TFIIF, or that TFIIF was not involved in transcriptional inhibition of the CMV and TK promoters. The study in which N-mHtt was shown to associate with RAP30 suggested that N-mHtt inhibited transcription by sequestering RAP30 from RAP74 [30]. This theory would predict that N-mHtt would be absent from an affected promoter. A previous study demonstrated that the ability to detect RAP30 association at a highly active promoter using chromatin immunoprecipitation was lost following activation of transcription at that promoter [31]. Chromatin immunoprecipitation was performed several times to quantify RAP30 association with the CMV promoter in N548wt and N548hd cells, however no specific association was detected (data not shown). *In vitro* promoter binding assays were performed to determine whether N-mHtt was present in the complement of proteins directly associated with the N-mHtt-affected CMV and TK promoters. A biotinylated 297 bp fragment of the CMV promoter, and a 768 bp fragment of the TK promoter were attached to streptavadin-coated magnetic beads and incubated with nuclear extract from either N548wt or N548hd cells. The proteins specifically associated with the promoters were isolated and probed with an antibody specific for Htt. Bands were detected in the complement of proteins specifically associated with both the CMV and TK promoters at the sizes predicted for the N-terminal fragment of Htt overexpressed in N548wt and N548hd cells (Fig. 5). The absence of Htt-immunoreactive bands in wash #3 suggested that the N-terminal Htt detected in the bound sample was specifically associated with the promoters. This result suggested that N-mHtt may impair transcription driven by the CMV and TK promoters by associating with promoter-bound complexes and impairing their formation or function rather than by sequestering proteins from the promoter.

**Figure 6:**
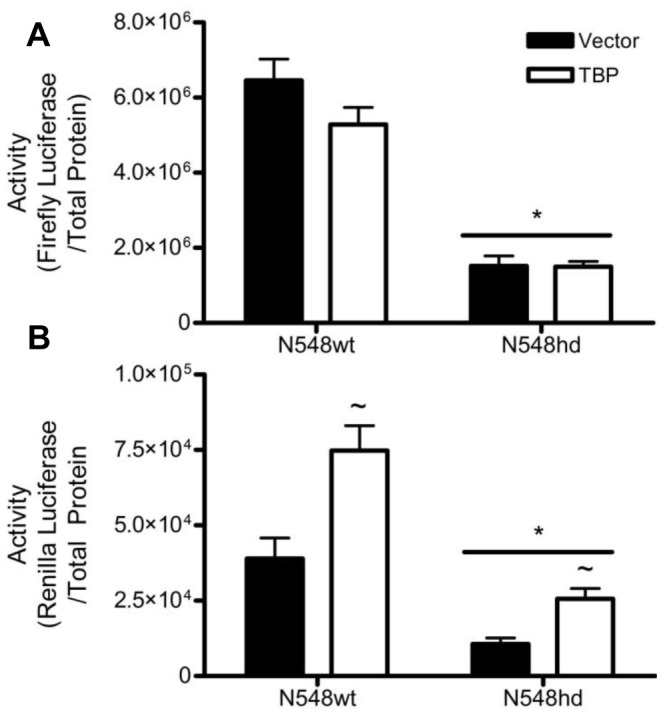
TBP overexpression increased transcription driven by the TK but not the CMV promoter in N548hd cells. N548wt and N548hd cells were transfected with CMV (A) and TK (B) reporter plasmids and either an empty expression plasmid, or one driving production of TBP cDNA. Luciferase activity was normalized to total protein. * *P<*0.05 relative to N548wt cells. ∼ *P<*0.05 relative to empty vector control within cell type as determined by two-way ANOVA followed by Bonferroni post-hoc analysis. Two-tailed *t-*tests were performed to analyze effect of TBP expression within cell type. Data are shown as mean ± S.E.M. n = 8 per data set.

The presence of N-mHtt in the complement of protein associated with two N-mHtt-affected promoters suggested that N-mHtt is recruited to an affected promoter. Mutation of candidate binding sites on the CMV promoter for transcription factors known to bind to N-mHtt did not restore transcription in N548hd cells, however that experiment did not directly manipulate the general transcription machinery. TBP is a member of the general transcription machinery that has been previously shown to interact with N-mHtt. To determine if N-mHtt impaired TK or CMV activity through interactions with TBP, plasmids driving the expression of human TBP cDNA, or its empty vector control, were transfected into N548wt and N548hd cells along with CMV and TK reporter plasmids. As was seen following overexpression of TFIIF, TBP overexpression failed to increase transcription of the CMV promoter in either N548wt or N548hd cells (Fig. 6). TK activity, on the other hand, was significantly increased in both N548wt and N548hd cells following TBP overexpression. As TBP is involved in all transcriptional events, it would be assumed that if TBP was involved in the mechanism of N-mHtt-mediated transcriptional dysregulation for one promoter, it would be involved in the mechanism for all promoters. The inability of TBP overexpression to restore CMV activity in N548hd cells suggested either that an additional mechanism is involved in inhibition of CMV activity, or that N-mHtt may impair transcription more severely at certain promoters, making it more difficult to overcome transcriptional inhibition.

**Figure 7:**
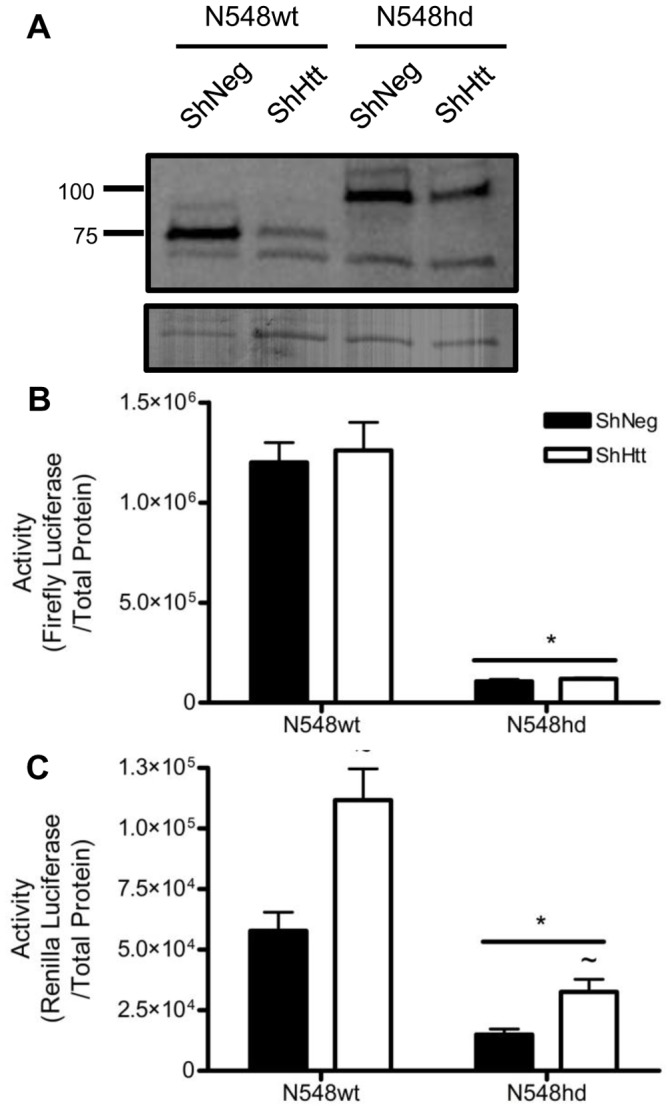
shRNA-mediated reduction of N-mHtt increased transcription driven by the TK but not the CMV promoter in N548hd cells. (A) N548wt and N548hd cells were transfected with a plasmid driving expression of shRNA complementary to nt 413–436 of *huntingtin* mRNA (shHtt) or an empty vector control (ShNeg). Cell lysates were collected and western blotting was performed using an antibody specific to Htt. Amido staining of the membrane is shown below. Numbers on the left represent the relative mobility of molecular weight size markers in kDa. N548wt and N548hd cells were transfected with CMV (B) and TK (C) reporter plasmids and either shHtt or shNeg plasmid. Luciferase activity was normalized to total protein. * *P<*0.05 relative to N548wt cells. ∼ *P<*0.05 relative to shNeg within cell type as determined by a two-way ANOVA followed by a Bonferroni post-hoc test. Two-tailed *t-*tests were performed to determined cell-specific effect of knock-down. Data are shown as mean ± S.E.M. n = 6 per data set.

To determine if a relationship existed between the amount of N-mHtt expression in N548hd cells and transcriptional inhibition of the CMV and TK promoters, shRNA-mediated knockdown was performed. Plasmids driving the expression of a shRNA sequence complementary to nt 413–436 of human *huntingtin* mRNA (shHtt) or its empty vector control (shNeg) were transfected into N548wt and N548hd cells along with reporter plasmids driven by the CMV and TK promoters. Transfection with shHtt reduced but did not eliminate N-terminal Htt expression in N548wt and N548hd cells (Fig. 7A). The reduction in N-terminal Htt expression had no effect on transcription driven by the CMV promoter in either N548wt or N548hd cells (Fig. 7B). Htt knockdown did, however, significantly increase transcription driven by the TK promoter in both N548wt and N548hd cells (Fig. 7C). This result suggested that CMV and TK promoter activity were differentially susceptible to the concentration of N-mHtt.

**Figure 8:**
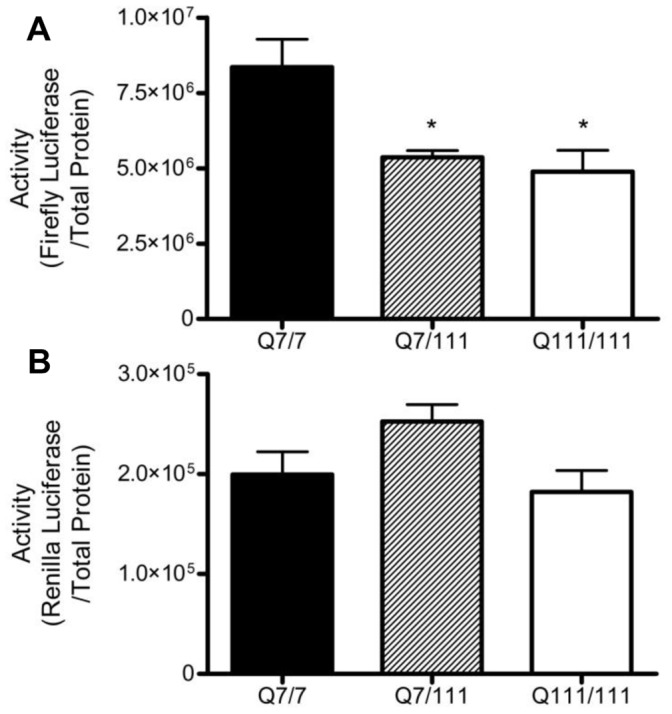
CMV but not TK promoter activity was decreased in ST*Hdh* Q7/111 and Q111/111 compared to Q7/7 cells. Reporter plasmids driven by the CMV (A) and TK (B) promoters were transfected in StHdh Q7/7, Q7/111, and Q111/111 cells. Cell lysates were collected 24 h post-transfection and a DLR™ Assay was performed. * P<0.05 relative to ST*Hdh* Q7/7 as determined by a one-way ANOVA followed by a Bonferroni post-hoc test. Data are shown as mean ± S.E.M. n = 8 for each data set.

A drawback of the N548 cell model is that the N-terminal fragment is highly overexpressed relative to levels observed in cells expressing full-length mutant huntingtin [32]. The amount of N-terminal Htt produced in N548 cells is under the control of the viral LTR promoter, and not the *huntingtin* promoter. The ST*Hdh* cell lines were obtained to test the susceptibility of the CMV and TK promoters to physiological levels of Htt. Reporter plasmids driven by the CMV and TK promoters were co-transfected into ST*Hdh* Q7/7, Q7/111, and Q111/111 cells and transcriptional activity was assayed 24 h following transfection. Similar to what was seen following shRNA-mediated knockdown in the N548 cell lines, CMV, but not TK, activity was inhibited in the presence of mHtt (Fig. 8). This result reinforces the theory that different promoters have differential susceptibilities to mHtt. The TK promoter, being less sensitive to the concentration of N-mHtt than the CMV promoter required a higher level of N-mHtt in the cell to inhibits its transcription. Specifically, TK activity was unaffected at the level of mHtt expression produced in ST*Hdh* cells and in N548hd cells following shRNA knockdown but was impaired in N548hd cells. The CMV promoter, being more sensitive to mHtt expression, had decreased transcriptional activity in the presence of each of the three amounts of N-mHtt tested.

**Figure 9:**
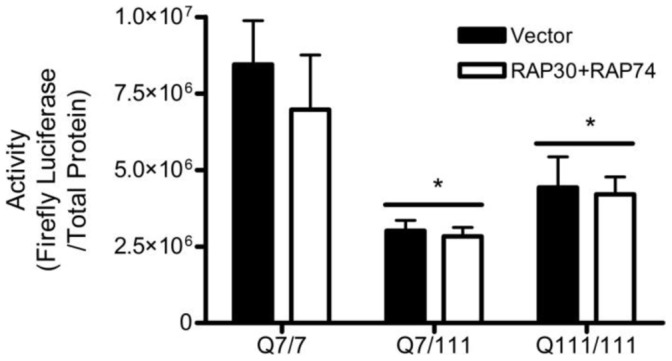
Overexpression of the components of TFIIF did not recover N-mHtt-mediated transcriptional repression. ST*Hdh* Q7/7, Q7/111 and Q111/111 cells were transfected with reporter plasmids driven by the CMV promoter and either an empty expression plasmid or ones driving production of both RAP30 and RAP74 protein. Luciferase activity was normalized to total protein. * P<0.05 relative to ST*Hdh* Q7/7 cells. *∼ P<*0.05 relative to vector control within cell type as determined by a two-way ANOVA followed by a Bonferroni post-hoc test. Two-tailed *t-*tests were performed to determined cell-specific effect of knock-down. Data are shown as mean ± S.E.M. n = 8 for each data set.

One possible reason why overexpression of TFIIF was unable to alleviate transcriptional repression of the CMV promoter in N548hd cells is that the level of N-mHtt expression was too great to overcome. To determine if overexpression of TFIIF could alleviate repression of the CMV promoter in the presence of physiological levels of mHtt, RAP30 and RAP74 were overexpressed in ST*Hdh* cells. Overexpression of the components of TFIIF failed to increase CMV activity in either the Q7/111 or Q111/111 cells (Fig. 9). Based on these results, it appears that either TFIIF is not involved in N-mHtt-mediated transcriptional repression of the CMV promoter or that N-mHtt associates with TFIIF in a way that cannot be overcome using RAP30 and RAP74 overexpression.

**Figure 10:**
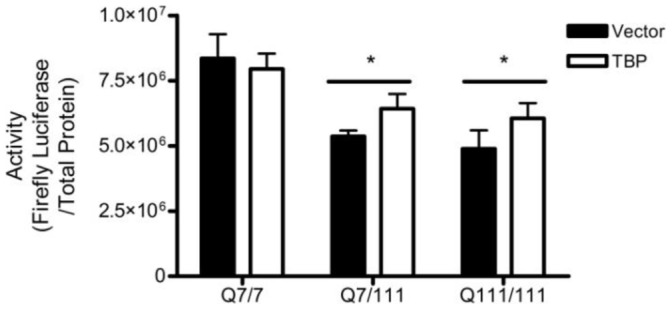
TBP overexpression did not recover N-mHtt-mediated transcriptional repression. ST*Hdh* Q7/7, Q7/111, and Q111/111 cells were transfected with a CMV reporter plasmid and either an empty expression plasmid, or one driving production of TBP cDNA. Luciferase activity was normalized to total protein. * *P<*0.05 relative to ST*Hdh* Q7/7 cells as determined by a two-way ANOVA followed by a Bonferroni post-hoc test. Two-tailed *t-*tests were performed to determined cell-specific effect of knock-down. Data are shown as mean ± S.E.M. n = 8 per data set.

Overexpression of TBP was able to alleviate transcriptional repression of the TK but not the CMV promoter in N548hd cells, leading to the assumption that different promoters may have different susceptibilities to the amount of N-mHtt in a cell, and consequently that it might be easier to alleviate transcriptional repression at some promoters than at others. We hypothesized that since mHtt expression is lower in ST*Hdh* cells than in the N548 cells, alleviating transcriptional repression of the CMV promoter might be more feasible in this cell model. Plasmids driving the expression of human TBP cDNA were transfected into the ST*Hdh* cell lines along with a reporter plasmid under the control of the CMV promoter. Overexpression of TBP did not significantly increase CMV activity in either the heterozygous or homozygous mHtt-expressing cells compared to cells transfected with empty vector control (Fig. 10). This result reinforces the observation that although transcription driven by the TK and CMV promoters are decreased by the same proportion in N548hd cells, the TK and CMV promoters have differential susceptibility with respect to the concentration of N-mHtt, and transcriptional repression is more easily alleviated at the less sensitive TK promoter than at the more susceptible CMV promoter.

## Discussion

The purpose of this study was to test and expand upon the current theories of N-mHtt-mediated transcriptional dysregulation using the highly active CMV and TK promoters. A promoter deletion experiment was performed to determine if a specific region of the CMV promoter was required for N-mHtt-mediated transcriptional repression. Although sequential deletion of the CMV promoter resulted in a progressive decrease in transcriptional activity, transcription driven by the smallest promoter fragment tested was significantly lower in N548hd cells compared to N548wt cells. Similar results have been reported in studies that utilized other N-mHtt-affected promoters. The minimally active fragments of the DARPP-32 (160 bp) [33] and PDE10A (69 bp) [34] promoters tested in N548wt and N548hd cells, and the smallest fragment of the adenosine A_2A_ receptor promoter (105 bp) [35] tested in PC12 cells inducibly expressing exon 1 of *huntingtin* with either 25 or 109 CAG repeats had decreased transcription in the presence of N-mHtt. These results suggest that N-mHtt may impair transcription through a mechanism involving interaction with proteins that bind near, or at, the transcription start site.

Linker-scanning mutagenesis was performed to determine if transcriptional repression of the CMV promoter was the result of interference by N-mHtt with the function of gene-specific transcription factors. Elimination of putative binding sites of transcription factors known to interact with N-mHtt did not restore CMV activity in N548hd cells to levels observed in N548wt cells. This suggested that no known N-mHtt-interacting transcription factor was solely responsible for inhibition of CMV activity. A previous study successfully alleviated N-mHtt-mediated transcriptional repression using linker-scanning mutagenesis. Elimination of a C/EBP binding site resulted in the recovery of transcription at the argininosuccinate acid lyase (AAL) promoter in the presence of N-mHtt [36]. A C/EBP binding site was eliminated from the CMV promoter in the present study, however no recovery in transcription was observed. This suggested that although N-mHtt is able to impair C/EBP-mediated transcriptional activation, this mechanism did not explain the full extent of transcriptional impairment of the CMV promoter. The findings that N-mHtt consistently impairs transcription driven by the minimally active fragment of affected promoters, and that elimination of binding sites for known N-mHtt-interacting transcription factors together suggest that N-mHtt may impair transcription through interactions with many components of the core transcriptional machinery.

TFIIF (composed of RAP30/RAP74 subunits) is a member of the core transcriptional machinery. RAP30 has been shown to interact with N-mHtt [30]. This interaction was shown to occur at the region of RAP30 required for interaction with RAP74. Overexpression of RAP30 and RAP74, either alone or in combination, was performed in an attempt to restore the soluble pool of the N-mHtt-interacting protein, to compete with N-mHtt for association with RAP30, and to increase the amount of functional TFIIF present in the cells. CMV and TK activity were not restored in N548 or ST*Hdh* cell lines expressing mHtt following overexpression of TFIIF or its components. This result suggested either that TFIIF was not involved in N-mHtt-mediated transcriptional repression of the CMV and TK promoters, or that N-mHtt was interacting with TFIIF in a manner that could not be overcome by overexpression of TFIIF or its components.

Numerous studies have suggested that N-mHtt interferes with transcription by binding to and sequestering gene-specific transcription factors from affected promoters [29], [30], [37]. Other studies have proposed that N-mHtt impairs transcription by interfering directly with the transcriptional machinery through associations with the DNA [38], [39]. An *in vitro* promoter binding assay was performed to determine whether N-mHtt was present or absent from the complement of proteins directly associated with the CMV promoter. In contrast to the predictions made by sequestration models of N-mHtt-mediated transcriptional dysregulation, N-mHtt in nuclear extract obtained from N548hd cells was among the proteins specifically associated with the N-mHtt-affected CMV and TK promoters. It is not possible to determine based on the technique used whether N-mHtt was bound directly to the DNA, or whether the presence of N-mHtt in the promoter-bound protein fraction resulted from its association with DNA-bound transcription factors or co-factors. Knowing that N-mHtt is capable of interacting with numerous proteins that make up the core transcriptional machinery [30], [24], [26], we would assume that N-mHtt is capable of inhibiting transcription through interactions with DNA binding proteins at the promoter. Consequently, a mechanism other than that proposed by the sequestration model is possible.

Although TFIIF did not appear to be involved in transcriptional repression of the CMV and TK promoters, evidence remained to suggest that N-mHtt impaired transcription through interactions with the core transcriptional machinery. TBP is another member of the core machinery and has been previously shown to interact with N-mHtt. TBP was overexpressed to determine whether it played a role in the decreased activity of the CMV and TK promoters in N548hd cells. TBP overexpression was able to significantly increase activity of the TK but not the CMV promoter in N548hd cells. As TBP is a key component of the basal transcriptional machinery, the involvement of TBP in N-mHtt-mediated transcriptional repression at one gene would suggest it contributes to some degree of transcriptional repression at all genes. The ability of TBP overexpression to alleviate transcriptional repression at the TK but not the CMV promoter suggested either that an additional mechanism was involved in inhibition of CMV activity, or that through a similar mechanism, N-mHtt exhibited a stronger inhibition of CMV activity than TK activity.

Genes are generally regarded as affected or unaffected with respect to their susceptibility to N-mHtt-mediated transcriptional repression. The possibility that the CMV and TK promoters had different degrees of susceptibility to N-mHtt was an interesting idea, and one that required further investigation. shRNA-mediated knockdown of N-mHtt was performed in N548hd cells to determine if transcriptional repression of the CMV and TK promoters could be alleviated by reducing the amount of N-mHtt in the cell. Reduction of N-mHtt expression increased transcription driven by the TK, but not the CMV, promoter in N548hd cells. This provided further evidence that two N-mHtt-affected promoters may have differential susceptibility to the effects of N-mHtt. The TK promoter, representing a more resistant promoter, was unaffected in the presence of the new, lower level of N-mHtt expression generated following shRNA expression. The CMV promoter, representing a more sensitive N-mHtt-affected promoter, remained susceptible to N-mHtt-mediated transcriptional repression at the new, lower N-mHtt expression level. Because the N548 cell model does not represent a physiologically accurate model of HD, the impact of mHtt on CMV and TK activity was tested using the ST*Hdh* cell model. Transcription driven by the CMV, but not the TK, promoter was significantly lower in both Q7/111 and Q111/111 cells compared to activity in Q7/7 wild-type cells. In agreement with the results from the shRNA knockdown experiment, the TK promoter, which models a more resistant N-mHtt-affected promoter, was unaffected at the lower level of N-mHtt expression in the StHdh cells, whereas the more sensitive CMV promoter exhibited decreased transcriptional activity. These two experiments provided further evidence that the CMV and TK promoters had differential susceptibility to the inhibitory effects of N-mHtt.

The inability of TFIIF or TBP overexpression to restore CMV activity in N548hd cells may have been due to the artificially high expression level of N-mHtt in this cell line. TFIIF and TBP were overexpressed in ST*Hdh* cells along with the CMV reporter plasmid to determine if CMV activity could be restored in cells expressing physiologically accurate levels of mHtt. Increasing expression of TFIIF or TBP did not increase transcription driven by the CMV reporter plasmid in the Q7/111 or Q111/111 cells relative to cells transfected with empty vector control. This result suggested that alleviating N-mHtt-mediated transcriptional repression at certain promoters is more complex than simply increasing the pool of soluble N-mHtt interacting proteins.

The primary conclusions of this study were that N-mHtt appeared to impair transcription near, or at the transcription start site, and that transcriptional repression may result from interactions between N-mHtt and DNA-binding transcription factors or co-factors occurring at the promoter. The differential susceptibility of the CMV and TK promoters to the concentration of N-mHtt present in striatal cells highlighted the fact that not all promoters are similarly affected. Increased expression and accumulation of mHtt, as well as increased nuclear accumulation of N-mHtt in animal models of HD have been correlated with disease onset and increased severity, suggesting that the symptoms of HD are directly associated with the concentration of mHtt [11], [40]. Caspase-mediated cleavage of mHtt has been shown to positively regulate caspase activity, suggesting that the concentration of nuclear N-mHtt increases as the disease progresses [41]. Of the genes that display decreased transcription during the progression of HD, some are affected earlier than others [20]. Based on our findings, the CMV promoter may model genes such as cannabinoid receptor 1, cyclic AMP phosphoprotein, and G protein-coupled receptor 6 which display the largest decreases in transcription early in the progression of HD [21]. Conversely, the TK promoter may model the numerous genes that are unaffected early in the progression of HD but display decreased transcription later in the disease when the nuclear concentration of N-mHtt is higher.

One important question that arises from these assumptions is, how are some genes more susceptible to the concentration of N-mHtt than others? One explanation is that transcription factors known to interact with N-mHtt may function as vehicles for recruiting N-mHtt to the promoter. Once recruited to the promoter, N-mHtt either impairs or inhibits the formation, release, or function of the core transcriptional complexes. Recruitment of N-mHtt to a promoter by a protein can be modeled in terms of probability. The probability, therefore, that transcription of a given gene will be impaired in the presence of N-mHtt is directly related to the concentration of N-mHtt present in the nucleus, the number of N-mHtt-interacting proteins required for proper transcription of that given gene, and their relative affinities for N-mHtt. This model assumes that N-mHtt can interact with multiple proteins simultaneously and that interaction inhibits the transcriptional machinery that facilitates normal transcription.

According to this model, promoters regulated by more N-mHtt-interacting proteins would recruit N-mHtt more frequently than promoters regulated by fewer N-mHtt-interacting proteins, and would display reduced transcript levels in the presence of lower concentrations of N-mHtt. This model would also explain why overexpression of a single N-mHtt-interacting protein would have limited efficacy in alleviating transcriptional repression at a promoter in the presence of N-mHtt. If a promoter is regulated by a large number of N-mHtt-interacting proteins, overexpression of one of those N-mHtt-interacting proteins would increase the non-N-mHtt-bound pool of that specific protein available for transcription, but would not change the frequency that the other N-mHtt-interacting proteins recruited N-mHtt to the promoter. In line with this assumption, very few studies have reported success in restoring transcriptional activity through overexpression of an individual N-mHtt-interacting protein. Conversely, two studies have shown success in overexpressing multiple transcription factors when expression of those proteins on their own proved unsuccessful [29], [41].

Several avenues exist to alleviate N-mHtt-mediated inhibition of transcription as proposed by this model. Overexpression of multiple N-mHtt-interacting proteins would be expected to have more success than overexpression of individual N-mHtt-interacting proteins at alleviating promoter-specific transcriptional represession. Overexpression of fragments of N-mHtt-interacting proteins that were able to associate with N-mHtt but not integrate into transcriptional complexes would have the added benefit of occupying N-mHtt and impeding its ability to associate with transcriptional complexes. Removing fragments of a promoter would likely reduce the number of N-mHtt-interacting proteins regulating the activity of that promoter because smaller promoters have fewer transcription factor binding sites than larger ones. Although this would reduce the vulnerability of a promoter to the effects of N-mHtt, it would remove endogenous regulatory mechanisms. While such strategies may work experimentally, the most therapeutically beneficial strategies would be those aimed at reducing the amount of N-mHtt in the nucleus, either through knockdown of mHtt, inhibiting the cleavage of mHtt, increasing nucleus efflux of N-mHtt or through increased degradation of the fragment. Each of these strategies would decrease the frequency with which N-mHtt was recruited to all promoters and in turn would reduce the frequency that N-mHtt inhibited transcription. Importantly, several groups have demonstrated that inhibition of mHtt cleavage decreases the concentration of N-mHtt in the nucleus and reduced cell death [8]–[11], [14], [42].

## Materials and Methods

### Cell Culture

Immortalized striatal cells, produced and originally characterized by Dr. Elena Cattaneo, from embryonic day 14 rats (ST14A), as well as derivatives stably expressing the N-terminal 548 amino acids of human Htt with 15 (N548wt), or 128 (N548hd) glutamine repeats (gifts from Dr. Elena Cattaneo), were cultured at the permissive temperature of 33°C with 5% CO_2_ and 95% air. The cells were grown in Dulbecco’s Modified Eagle Medium (DMEM, Gibco) supplemented with 10% (v/v) fetal bovine serum (FBS, Gibco) as described previously (Cattaneo and Conti, 1998). The generation and handling of ST14A cells and their derivatives was in accordance with the guidelines of the Institute of Pharmacological Sciences, University of Milano, Milan, Italy (Cattaneo and Conti, 1998).

ST*Hdh* Q7/7, Q7/111 and Q111/111 cells were purchased from the Coriell Institute for Medical Research. Cells were cultured at the permissive temperature of 33°C with 5% CO_2_ and 95% air in DMEM media as described in the literature provided by Coriell.

### Transfections & Luciferase Assay

Polymerase chain reaction (PCR) was performed to generate the CMV promoter sequences to create a CMV promoter reporter plasmid. The sense primer CTCGAGCCAGTGCCAAGCTGAT was used in combination with the antisense primer CACAGGACGGGTGTGGTC to amplify a 772 bp fragment of the CMV promoter using the pCMV-luc plasmid as a template. PCR reaction conditions consisted of a 15 m incubation at 95°C followed by 35 cycles of 95°C for 30 s, 58°C for 30 s, and 72°C for 1 m. The reaction was completed with a 10 m extension at 72°C. The truncated CMV promoter fragments were produced using the antisense primer above in combination with the sense primer GCCCAGTACATGACCTTACGGG (to produce the −227 CMV fragment) or AAATGTCGTAATAACCCCGCCC (to produce the −99 CMV fragment). The resulting PCR products were run on an agarose gel, gel extracted, cloned into pGEM-T vector and subsequently subcloned into *Mlu*I/*Bgl*II-digested pGL3-basic plasmid. Each of these steps was performed according to manufacturer’s directions.

**Table 1:** Primers used for linker-scanning mutagenesis.

Promoter Sequence	Orientation	Sequence
−277/266	Sense	ACGACGTACGAAGCCTTGGCAGTACATCT
	Antisense	GCTTCGTACGTCGTAAGGTCATGTAC
−172/161	Sense	ACGACGTACGAAGCGAGTTTGTTTTGGCACCA
	Antisense	GCTTCGTACGTCGTGGGGTGGAGACTTGGA
−149/136	Sense	ACGACGTACGAAGCGTCTCCACCCCATT
	Antisense	GCTTCGTACGTCGTGAGTCAAACCGCTAT
−109/98	Sense	ACGACGTACGAAGCAATGTCGTAATAACC
	Antisense	GCTTCGTACGTCGTTGATTTTGGTGCCAAAA
−109/71	Sense	ACGACGTACGAAGCAATGTCGTAATAACC
	Antisense	GCTTCGTACGTCGTGTTATTACGACATTTTG
−38/20	Sense	ACGACGTACGAAGCGCTCGTTTAGTGAACCGTCAGA
	Antisense	GCTTCGTACGTCGTCCTCCCACCGTACACGCCTA
−14/1	Sense	ACGACGTACGAAGCTCAGATCTGGTACCCAG
	Antisense	GCTTCGTACGTCGTCTAAACGAGCTCTGCTTA

Plasmids used in this study that were not generated in the lab included pEGFP-N1 (U55762 BD Biosciences), pRL-TK (AF025846, Promega), pCMV-luc (a gift from Dr. Mark Nachtigal), pCDNA-RAP30 and pCDNA-RAP74 (gifts from Dr. Dimitrius Kranic) and pProEx-Hta-TBP (a gift from Dr. Ulrich Hartl).

Cells were transfected with 50 ng pEGFP-N1 plasmid, 50 ng of pRL-TK plasmid, and 200 ng of pGL3-CMV plasmid using Lipofectamine (Invitrogen) and PLUS reagent (Invitrogen) according to manufacturer’s directions. In overexpression experiments, 200 ng of expression vector, or the equivalent mass of empty-vector control was added to the mix. Transfections were performed in serum-free media. Serum was added to the media to a final concentration of 10% (v/v) 4 h after transfection mixes were added to the cells.

Twenty-four hours after transfection, promoter activity was measured using the DLR™ Assay System (Promega) according to manufacturer’s protocol using a 20/20^n^ luminometer (Turner Biosystems). Following quantification of TK and CMV activity, the protein concentration in the collection sample was determined using a standard Bradford Assay.

### Linker-Scanning Mutagenesis

The 297 bp of the CMV promoter 5′ to the transcription start site were analyzed using MatInspector online software, provided by Genomatix software suite (version 8.0, 2009). Putative transcription factor binding sites identified by the online matrix for transcription factors that have been previously shown to interact with mHtt were targeted for deletion. The primer pairs described in Table 1 were used in combination with the sense primer CTCGAGCCAGTGCCAAGCTGAT and the antisense primer CACAGGACGGGTGTGGTC to produce CMV promoter fragments upstream and downstream of the sequence targeted for mutation. The resulting PCR products were cloned into pGEM-T vectors and then extracted using restriction digest. The two fragments were then ligated together using the complementary linker portion. PCR was used to amplify the ligated CMV promoter containing a mutated target sequence. The PCR product was isolated, ligated into pGEM-T, and then subcloned into a pGL3 reporter plasmid. DNA sequencing was performed to verify the presence and location of each mutation.

### *In Vitro* Promoter-Binding Assay

A 3′ biotinylated fragment of the CMV promoter spanning from 297 bp 5′ to 75 bp 3′, relative to the transcription start site was generated via PCR from the pGL3-CMV reporter plasmid using primer sequences GCCCAGTACATGACCTTACGGG and CTTTATGTTTTTGGCGTCTTCC. A 3′ biotinylated fragment of the TK promoter spanning from 768 bp 5′ to 27 bp 3′, relative to the transcription start site was generated via PCR from the pRL-TK reporter plasmid using primer sequences CGGTGGTTAGGGTTTGTCTGACGC and GCAGGGTCGCTCGGTGTTCG. The promoter fragments were attached to streptavadin-coated magnetic M-280 dynabeads (Invitrogen) using a MPC®-S Magnetic Particle Concentrator (Dynal Biotech) according to manufacturer protocol. Prior to the binding assay, the DNA-bound beads were incubated for 15 min in 150 µl blocking buffer [100 mM KCl, 20 mM HEPES (pH 7.6) (Sigma), 5 mM MgCl_2_ (Sigma), 2 mM EDTA, 60 mg/ml casein (Sigma), 5 mg/ml polyvinylpyrrolidine (Sigma), 2.5 mM DTT] and then washed with transcription buffer (see manufacturers protocol).

Nuclear extract from N548wt and N548hd cells was extracted using the NE-PER Nuclear and Cytoplasmic Extraction Reagents (Pierce) according to kit protocol, and the protein was quantified using a standard Bradford assay. Nuclear extract from N548wt and N548hd cells was combined with binding buffer [100 mM KCl, 20 mM HEPES (pH 7.6), 5 mM MgCl_2_, 2 mM EDTA, 2.5 mM DTT, 0.05% (v/v) NP40, 30 ng/µl poly dI:dC] to a final volume of 50 µl and added to the Dynabead-bound promoters. The tubes were incubated at room temperature on the rotisserie shaker for 30 m. The tubes were placed on the magnet for 2 m and the supernatant was removed and stored as the unbound fraction. The pellets were washed 3 times with 400 µl reaction wash buffer [100 mM KCl, 20 mM HEPES (pH 7.6), 5 mM MgCl_2_, 2 mM EDTA, 2.5 mM DTT, 0.05% NP-40] and the supernatants were collected for analysis. The final pellet was suspended in 20 µl of Laemmli Sample Buffer (BioRad), fractionated on an polyacrylamide gel, and was probed with antibody MAB2166.

### shRNA-Mediated Knockdown

N548wt and N548hd cells were transfected with a mix containing 200 ng of pGL3-CMV plasmid, 50 ng of pRL-TK plasmid, 50 ng of pEGFP-N1 plasmid and either 30 nM plasmid driving the expression of RNA complementary to the sequence between nucleotides 413–438 of human *huntingtin* mRNA (shHtt) or empty plasmid (shNeg) as described previously. The cells were grown for 48 h following transfection, at which point cell lysates were collected and the DLR™ Assay was performed as described. Western blotting was performed using antibody MAB2166 to probe a gel containing 5 µg of cell lysate.

### Antibodies Used

Antibodies used in this study included anti-huntingtin antibody MAB2166 (Millipore), anti-RAP30 antibody ab28180 (abcam) and anti-RAP74 antibody ab28179 (abcam).

### Statistical Analyses

Statistical significance was set at 0.05 and determined via one- or two-tailed *t*-test or one- or two-way ANOVA as indicted in each figure legend. Two-way ANOVA were followed by Tamhane’s T2 post-hoc test for unequal variance or Bonferroni post-hoc test as indicated. Data are shown as the mean ± the standard error of the mean (S.E.M.).
